# Vascularization and odontode structure of a dorsal ridge spine of *Romundina stellina* Ørvig 1975

**DOI:** 10.1371/journal.pone.0189833

**Published:** 2017-12-27

**Authors:** Anna Jerve, Qingming Qu, Sophie Sanchez, Per Erik Ahlberg, Tatjana Haitina

**Affiliations:** 1 Biology Department, Imperial College London, Silwood Park Campus, Ascot, United Kingdom; 2 Department of Organismal Biology, Uppsala University, Uppsala, Sweden; 3 Centre for Advanced Research in Environmental Genomics, University of Ottawa, Ottawa, Ontario, Canada; 4 Science for Life Laboratory and Uppsala University, Department of Organismal Biology, Uppsala, Sweden; 5 European Synchrotron Radiation Facility, Grenoble, France; Indiana University Bloomington, UNITED STATES

## Abstract

There are two types of dermal skeletons in jawed vertebrates: placoderms and osteichthyans carry large bony plates (macromery), whereas chondrichthyans and acanthodians are covered by small scales (micromery). Fin spines are one of the last large dermal structures found on micromeric taxa and offer a potential source of histology and morphology that can be compared to those found on macromeric groups. Dermal fin spines offer a variety of morphology but aspects of their growth modes and homology are unclear. Here, we provide detailed descriptions of the microstructure and growth of a dorsal ridge spine from the acanthothoracid placoderm, *Romundina stellina*, using virtual three-dimensional paleohistological datasets. From these data we identify several layers of dentine ornamentation covering the lateral surfaces of the spine and reconstructed their growth pattern. We show that this spine likely grew posteriorly and proximally from a narrow portion of bone located along the leading edge of the spine. The spine is similarly constructed to the scales with a few exceptions, including the absence of polarized fibers distributed throughout the bone and the presence of a thin layer of perichondral bone. The composition of the spine (semidentine odontodes, dermal bone, perichondral bone) is identical to that of the *Romundina* dermal plates. These results illustrate the similarities and differences between the dermal tissues in *Romundina* and indicate that the spine grew differently from the dentinous fin spines from extant and fossil chondrichthyans. The morphology and histology of *Romundina* is most similar to the fin spine of the probable stem osteichthyan *Lophosteus*, with a well-developed inner cellular bony base and star-shaped odontodes on the surface. Results from these studies will undoubtedly have impact on our understanding of fossil fin spine histology and evolution, contributing to the on-going revision of early gnathostome phylogeny.

## Introduction

Macromeric skeletons are characterized by large, morphologically distinctive and phylogenetically conserved dermal bones covering the head and shoulder girdle [1]. Typically, individual dermal bones can be homologized across large clades: for example, the maxilla, premaxilla and dentary can be uncontroversially identified–or, in a few cases, shown to be absent–in all members of the Osteichthyes [2,3]. A macromeric skeleton usually consists of segmentally arranged scales of dermal bone, which sometimes carry odontodes and are pierced by sensory line canals. Micromeric skeletons do not contain large dermal bones and the fundamental unit of this type of skeleton is a small dermal scale, which extends as a relatively homogeneous squamation across the whole animal [1]. The scales almost always have a dentine crown; the base may consist of bone, osteodentine or dentine [4]. Among extant jawed-vertebrates, macromery occurs in osteichthyans (bony fishes and tetrapods) whereas micromery is found in chondrichthyans (cartilaginous fishes). Two extinct groups of Palaeozoic jawed vertebrates, the placoderms and acanthodians, are characterized, respectively, by macromeric and micromeric skeletons.

The status of living osteichthyans and chondrichthyans as monophyletic sister groups is generally well supported by morphology and molecular data [5,6]. However, the relationships of both placoderms and acanthodians have undergone major reevaluations in recent years and they are now generally regarded as paraphyletic arrays of taxa within the gnathostome (jawed vertebrate) and chondrichthyan stem groups, respectively [2,3,7,8,9,10,11, but see also [12] in support for placoderm monophyly]. This hypothesis received strong support from the discovery of *Entelognathus* and *Qilinyu*, two Silurian vertebrates from Yunnan, China, that combine a placoderm-like dermal skeleton with typical osteichthyan marginal jawbones [2,3]. These data offer support to the present view that the macromeric dermal skeletons of placoderms and osteichthyans are homologous, placing all micromeric gnathostomes within the chondrichthyan total group.

Fin spines are, with very few exceptions, the only large dermal skeletal elements in micromeric jawed-vertebrates. They are widely distributed among acanthodians, in which they are normally present on all fins except the caudal [10,13]. In conventional chondrichthyans they typically only occur on the dorsal fins, although paired fin spines have also now been described in some Devonian forms [14]. The likely position of acanthodians in the chondrichthyan stem group, below conventional chondrichthyans, suggests that paired fin spines are primitively present in total-group Chondrichthyes but have been lost in more derived members of the group. Among extant chondrichthyans they occur in all holocephalans and a small number of sharks [15,16].

Spine-shaped dermal elements also occur at the leading edges of some fins in certain primitive fossil osteichthyans such as *Psarolepis* and *Guiyu* [17,18]. In some placoderms, including *Romundina*, a symmetrical midline spine is sometimes present as an expansion of the posterior median dorsal plate [19]. Modified versions of this spine can also be found in some ptyctodonts and arthrodires [20,21]. However, it is far from certain that all these spines and spine-like structures are homologous. Their tissue composition is known to vary considerably, especially as concerns the presence or absence of bone [10,16,22,23,24].

*Romundina stellina* is an acanthothoracid-grade placoderm from the Early Devonian (Lochkovian) of arctic Canada [25]. It is considered by some as one of the most basal placoderms and has been integral for studying questions about the primitive gnathostome condition [19,26,27,28,29]. While this position has been challenged recently [12], understanding all available skeletal elements from any placoderm remains important, as very little is known about the postcranial skeleton of placoderms in general. These elements could thus provide valuable information about the evolution of the macromeric dermal skeleton in early jawed vertebrates.

Studies have shown that placoderm skeletons can have a distinct and complex histology, with the details shown by using non-destructive synchrotron technology to study the microstructure of the dermal skeleton [30]. Some of these histological structures, for example, the vascular mesh or the density of bone cell spaces, largely reflect the growth pattern of individual bony plates [24,26,28]. Here, we present a three-dimensional histological study of a dorsal ridge spine from *Romundina stellina*, produced by synchrotron microtomography, with the dual aim of illuminating the histological organization of the dermal skeleton and understanding the growth of fin spines in early vertebrates. By investigating the three-dimensional architecture and growth mode of a dorsal spine of *Romundina stellina*, we aim to provide a basis for comparison not only with extant chondrichthyans in which fin spine growth has been studied directly [16], but also with acanthodians and extinct chondrichthyans that can be imaged the same way, and will provide new data to address the question of fin spine homology [31,32].

## Materials and methods

The *Romundina stellina* spine described here is catalogued in the collections of the Naturhistoriska Riksmuseet in Stockholm, Sweden, as NRM P.11714. It is associated with the holotype specimen [25] and other *R*. *stellina* material that was collected from Prince of Wales Island in Canada [26,29], and has likewise been chemically prepared using a weak formic acid solution (see Dupret et al. [26] for details).

Synchrotron datasets aimed at providing information on the vascularization and odontode structure were obtained using Propagation Phase-Contrast Synchrotron X-Ray Microtomography (PPC-SRμCT) at beamline ID19 of the European Synchrotron Radiation Facility (ESRF) in France (e.g. [24,33,34]). A multiscale approach was used to image the *Romundina stellina* spine with a voxel size of 7.46 and 0.678 μm. The low-resolution scan (voxel size: 7.46 μm) was performed using a monochromatic beam with an energy of 40 keV fixed with a double Si111 crystal monochromator in Bragg reflection. A 1.8 mm aluminum filter was used. The optical system was mounted with a 10 μm thick Gadox scintillator coupled to a Frelon CCD camera (fast readout low noise) [35]. The experiment was performed with 1999 projections over 360° in continuous rotation mode. The time of exposure was of 0.3 s. A propagation phase contrast distance of 900 mm was used to increase the contrast between the spine microstructures. The high-resolution scan (voxel size: 0.678 μm) was performed using a single crystal 2.5nm period W/B4C multilayer monochromator to obtain an energy of 30 keV. The U32u undulator’s gap was closed to 12.38 mm. The beam was filtered with 2 mm of aluminum. The optical system was coupled to a FreLoN 2K14 CCD camera (fast readout low noise) and a GGG10 scintillator [36]. 2000 projections were taken over 180° in continuous rotation mode with a time of exposure of 0.3 s. The sample was fixed at a propagation distance of 30 mm. These datasets were reconstructed using a filtered back projection algorithm (PyHST software, ESRF) in edge detection mode [33]. They were rendered and segmented using VG StudioMax 2.2 (Volume Graphics, Germany).

## Results

### Morphological description

**Fig 1:**
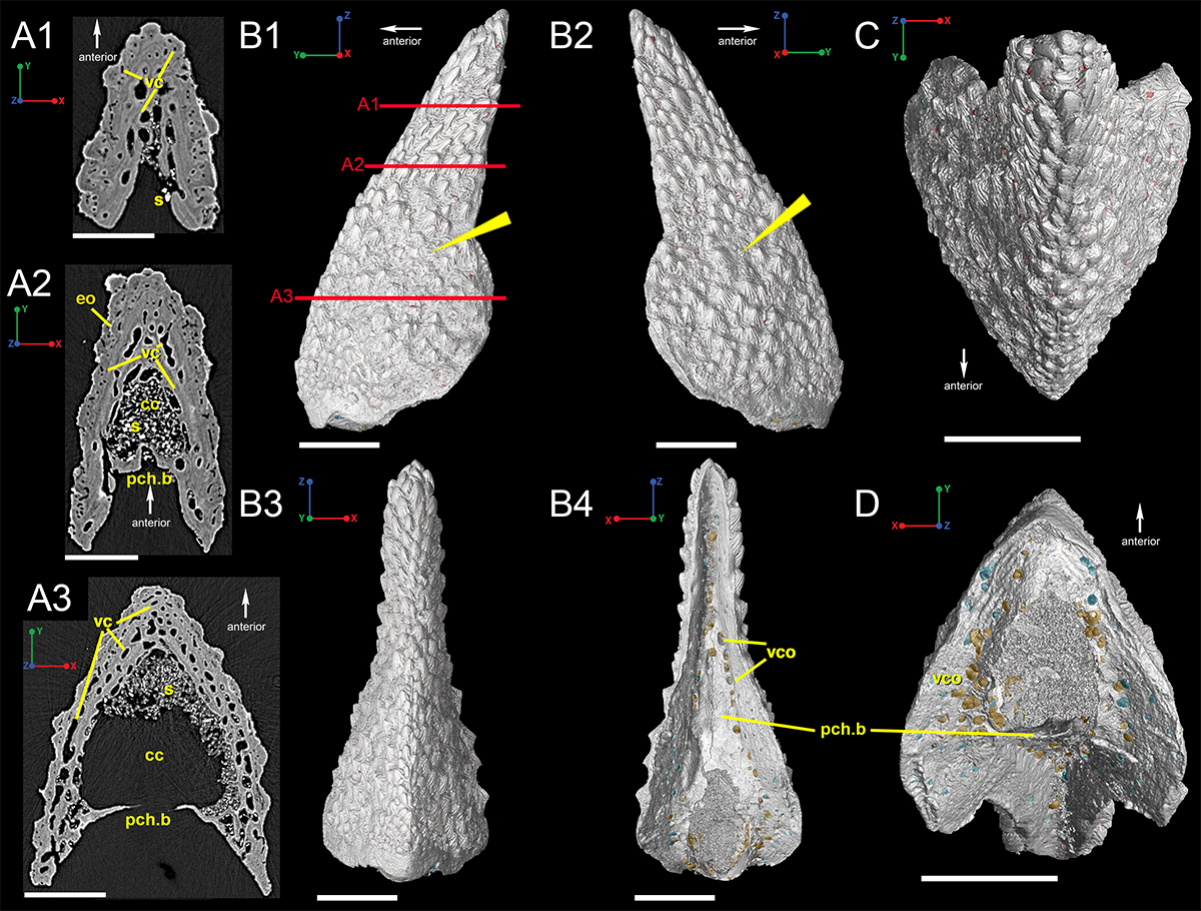
Virtual thin sections in transverse view of the dorsal ridge spine of *Romundina stellina* (P.11714). (A1-A3) Three-dimensional reconstruction created from low-resolution (7.46 micron) synchrotron data. Red lines on B1 indicate approximate locations of A1-A3. (B1-B4) 3D reconstructions of the spine illustrating its overall morphology in (B1) left lateral, (B2) right lateral, (B3) anterior, and (B4) posterior, (C) dorsal, and (D) ventral views. Scale bars are 1500 μm. Abbreviations are: cc, central canal; eo, embedded odontode; pch.b, perichondral bone; s, sediment; vc, vascular canal; vco, vascular canal opening.

Segmented scan data taken at 7.46 μm voxel size reveal the overall morphology of the dorsal spine of *Romundina* (Fig 1). The *Romundina* spine approximates an isosceles triangle in shape, with a broad base that tapers apically (Fig 1). The lateral sides of the spine intersect anteriorly to form the leading edge. The free edges of the lateral sides are not linear, but are instead flared laterally and posteriorly roughly halfway down the length of the spine (Fig 1B). This creates a rounded wing-like bump on each side of the spine when it is viewed laterally and a broom-like appearance when viewed posteriorly (Fig 1B).

### Histological description

**Fig 2:**
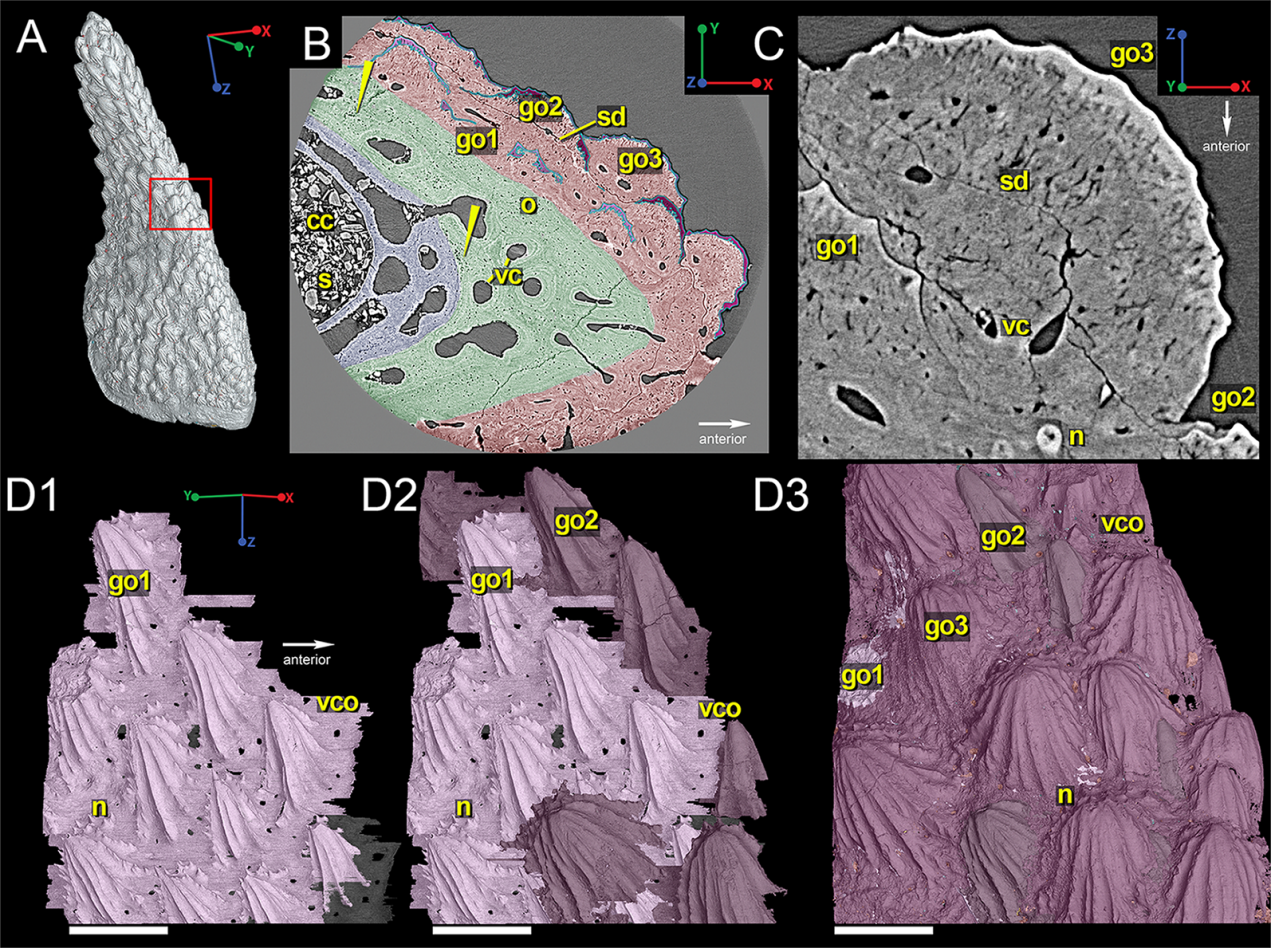
Right oblique view of the spine reconstructed from low-resolution data (7.46 μm). (A) Shows the approximate location of the area of interest examined at high-resolution (0.678 μm). Scale bar is 1500 μm. (B) Virtual thin section of the spine, showing its overall histological organization, with generations of odontodes highlighted in different shades of pink. Yellow arrows indicate the presence of poorly mineralized bone. Blue is bone with many vascular canals, green is bone with few vascular canals, and red is the outer layer of dentine ornamentation. Scale bar is 300 μm. (C) Closer view of an odontode. Scale bar is 70 μm. (D1-D3) 3D reconstruction of odontode morphology, showing relative distribution of odontodes in three generations (go1, pink; g0 2. dark purple; go3, light purple). Scale bar is 300 μm. Abbreviations are: cc, central canal; go1, 2, 3, first, second, third generation odontodes; n, node; o, osteocyte spaces; s, sediment; sd, semidentine; vc, vascular canal; vco, vascular canal opening.

Three different layers of hard tissue comprise the bulk of the spine: an inner bone layer with many vascular canals and few cell spaces (Fig 2B, blue); a middle bone layer with proportionately fewer vascular canals and many cell spaces (Fig 2B, green); and an outer semidentine ornament (odontodes; Fig 2B, red). Within the bone of the middle layer there are small clusters of cell spaces that could represent zones of poorly mineralized bone tissue (Fig 2B; yellow arrowheads).

A thin layer of presumably perichondral bone is present on the posterior side of the spine, connecting the two lateral sides (Fig 1A2, 1A3, 1B4 and 1D). This bone is thin and anterior to the furthest posterior edge of the lateral edges, giving the posterior side of the spine a convex geometry (Fig 1A2, 1A3 and 1B4; pch.b). The bone of the posterior side houses numerous vascular canal openings (Fig 1A1, 1B4 and 1D). A posteromedially positioned large ovoid opening lies within the perichondral bone and this leads into the central vascular area of the spine (Fig 1B4 and 1D). We interpret the space between the perichondral bone and the dermal bone of the spine as the central cavity (Fig 1A2 and 1A3; cc) for a supporting cartilage, somewhat similar to that in the dorsal fin spine of *Callorhinchus* [16]. The base of the spine is thin and unornamented (Fig 1B).

Odontodes are distributed across the lateral surfaces of the spine but there are certain areas on the lateral faces of the spine that are not heavily ornamented (Fig 1B1 and 1B2; arrows). The odontodes are stellate and composed of several ridgelets, with the largest being smooth and pointing apically (Figs 1 and 2). Near the base of the spine, the odontodes are more rounded in shape (Fig 1B2). Odontodes on the upper half of the spine seem to be aligned in semi-parallel rows that are angled outward from the leading edge. However, there is no specific set of odontodes that forms the leading edge (Fig 1C and 1B).

High-resolution scan data show that three generations of semidentine odontodes comprise the anterolateral surfaces spine (Fig 2). Each odontode has a thin and uneven outermost layer of what appears to be denser tissue (higher density is brighter in synchrotron scan slices) that is thickest in the tallest part of the odontode and thins out along the edges (Fig 2C). The oldest odontode generation (first generation, go1) has a common depositional surface, which is generally fully buried, resulting in these odontodes having the best-defined ridgelets and sharp nodules (Fig 2D1; pink). These odontodes are longer and narrower than younger generations. Lateral and posterior ridgelets from this generation (posterior to the apex of the main ridge of the odontode) can bear many sharp, pointed nodules, creating a serrated pattern. In contrast, anterior ridgelets from individual odontodes from this generation are usually smooth and bear no nodules. Second generation odontodes (go2) are partially buried, larger in size and have fewer serrations and the nodules are more rounded (Fig 2D2; dark purple). These also have a variety of different morphologies, with some being long and narrow and some rounder. The layer is not continuous over the first generation of odontodes, but seems to have its own depositional surface that is separate from those of the first generation odontodes. The youngest odontodes, called third generation odontodes (go3), share a depositional surface and creates the outer surface of the spine (Fig 2D3; light purple). These odontodes are larger and wider than the first- and second-generation odontodes and have only some rounded posterior nodes and no serrated ridgelets, probably due to postmortem erosion. Vascular canal openings cut through each of the depositional surfaces, ultimately opening to the surface of the spine (Fig 2).

**Fig 3:**
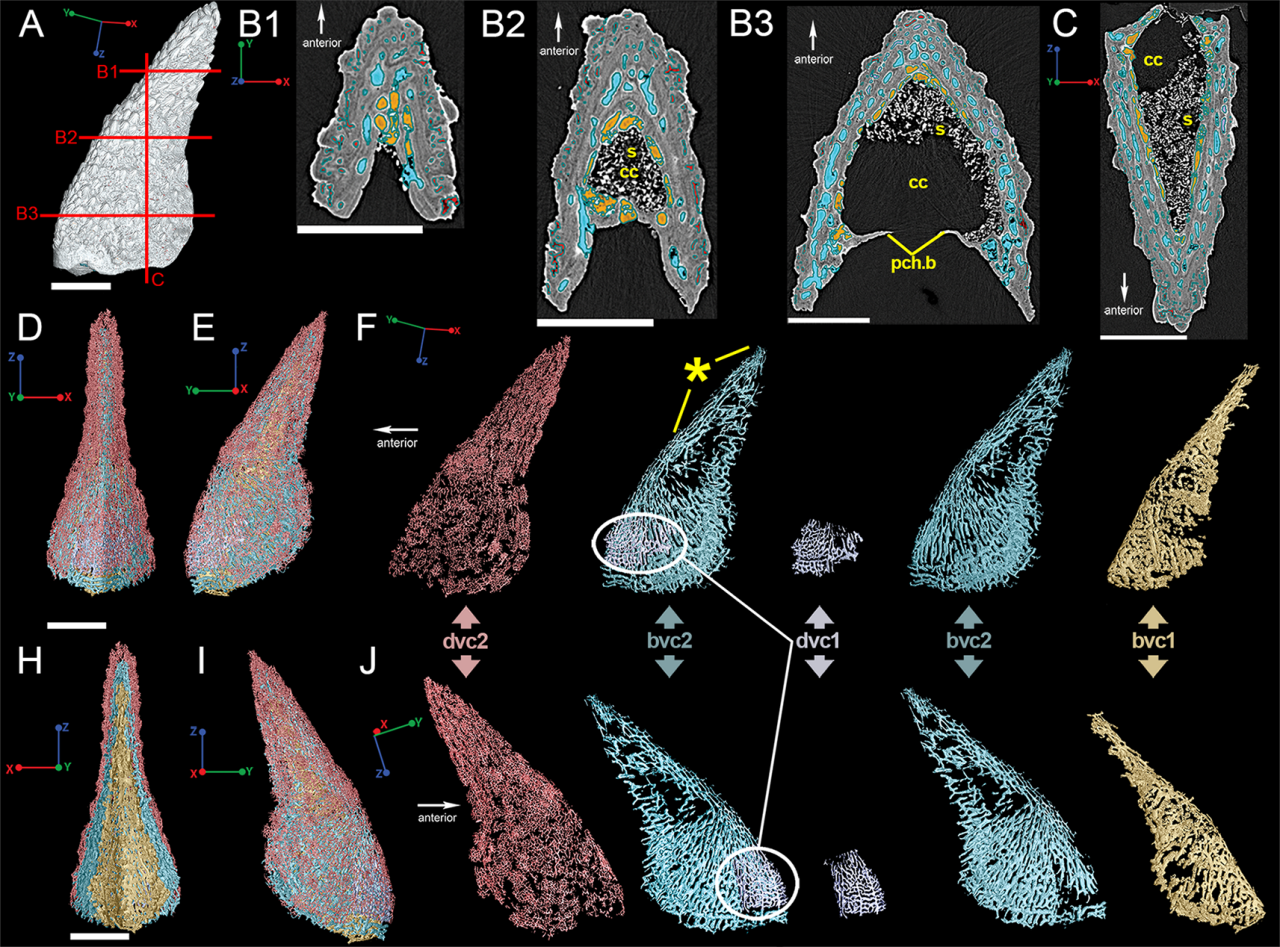
Vascularization of the *Romundina* dorsal ridge spine using low-resolution synchrotron data (7.46 μm). (A) Reconstruction of the dorsal spine showing approximate locations of slices B1-B3 and C. Scale bar is 2000 μm. Virtual thin sections taken through the spine on (B1-B3) transverse and (C) frontal planes. Scale bars 1500 μm. (D) Anterior and (E) left lateral views of the reconstructed vascularization in the spine, while (F) deconstructs the vascular canals into layers beginning with the outermost layer in red. These colors relate to the virtual thin sections in (B1-B3). Star indicates relative position of the spine's growth origin. (H) Posterior and (I) right lateral views of the spine with similar vascular break down to (F) but in right lateral view. Scale bars are 2000 μm. Abbreviations are: bvc, bone vascular canals; cc, central cavity; dvc, dentine vascular canals; pch.b, perichondral bone; s, sediment.

**Fig 4:**
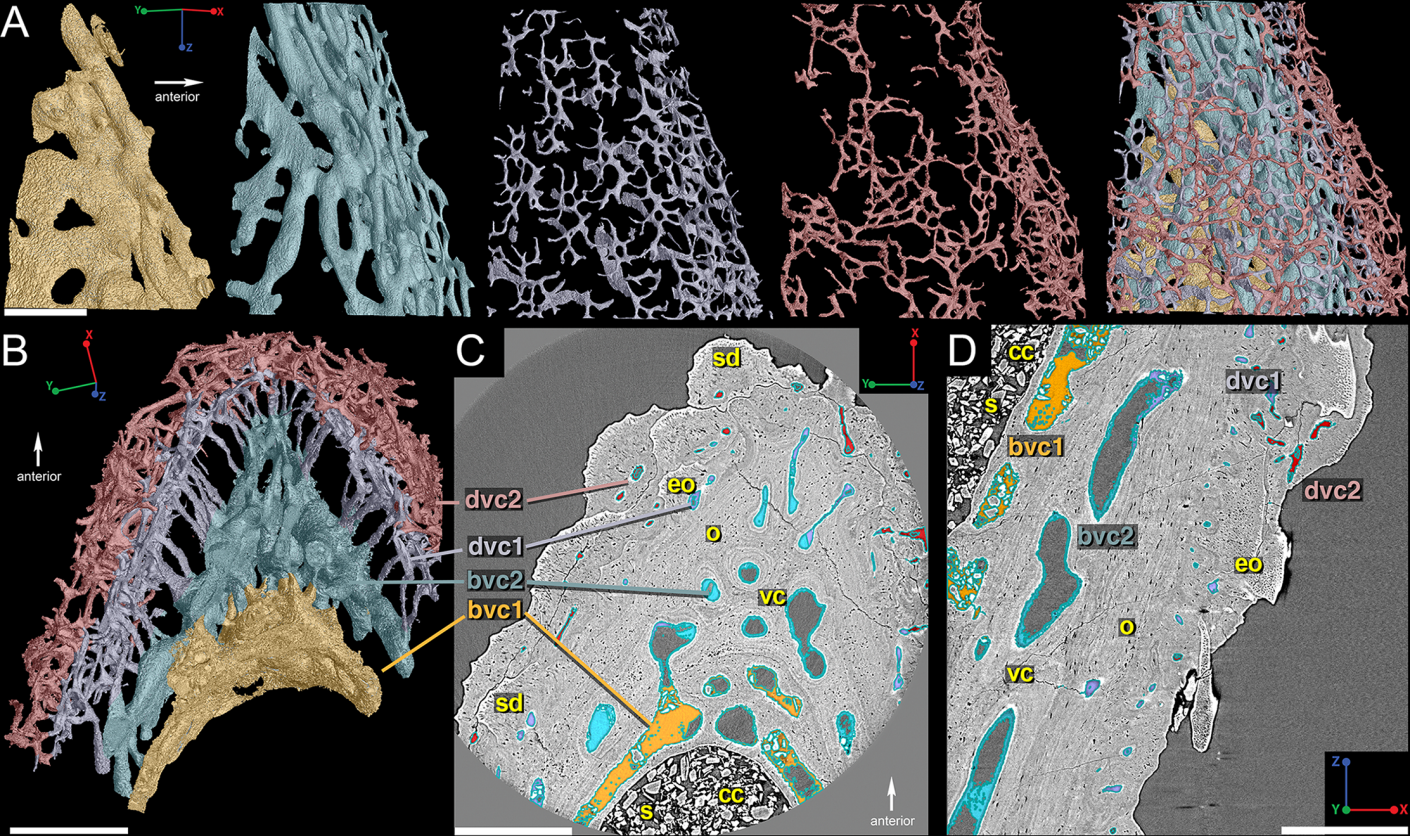
Vascularization of the *Romundina* dorsal ridge spine using high-resolution synchrotron data (0.678 μm). (A) 3D reconstructions of the four individual layers of vascular canals beginning with the innermost layer (yellow). Scale bar is 300 μm. (B) Virtual thin-section of the reconstructed vascularization that illustrates how the different layers of canals relate to each other. Scale bar is 350 μm. (C) and (D) Virtual thin sections with the different vascular layers highlighted to correspond to the reconstructed data. Scale bar is 350 μm. Abbreviations are: bvc, bone vascular canals; cc, central cavity; dvc, dentine vascular canals; eo, embedded odontode; o, osteocyte spaces; s, sediment; sd, semidentine; vc, vascular canal.

The reconstruction of the outermost layer of canals creates a pattern that follows the distribution of the dentine ornament (Fig 3F and 3J; red) while the second innermost layer (Fig 3F and 3J; blue) consists of longer sections of canals that have short connections to each other and suggests by its geometry that growth was occurring at the proximal and posterior margins of the bone, with the original "growth center" (i.e. the oldest part of the spine) being located along the distal part of the anterior margin (Fig 3F and 3J; star). The 3D reconstructions of the other layers of canals are either patchy or incomplete and difficult to interpret (Fig 3F and 3J; purple & yellow). High-resolution synchrotron scan data (voxel size 0.678 μm) of a region of interest (Fig 2A) near the leading edge of the spine provides a clearer picture of the vascular morphology (Fig 4). 3D reconstructions show that there are indeed four layers of canals; the innermost two layers correspond to bone deposition and are referred to as bone vascular canals (bvc) and the two outer layers relate to the dentine ornamentation and are designated dentine vascular canals (Fig 4B, 4C and 4D; dvc).

The first layer of bone vascular canals is generally large, wide, and flat (Fig 4; bvc1; yellow). These tend to be situated close to the central space that contained the supporting cartilage, usually separated from it by a thin layer of presumably perichondral bone (Fig 3B3). They thus represent a vasculature that is situated between the dermal bone and the cartilage core, rather than within the dermal bone itself. These canals merge into the second layer of bone vascular canals (Fig 4; bvc2; blue) via short vertical canals, which gives both layers a compact appearance (Fig 4B). The canals of bvc2 tend to be larger and flatter near bvc1 and thinner and rounder distally (Fig 4A and 4B).

**Fig 5:**
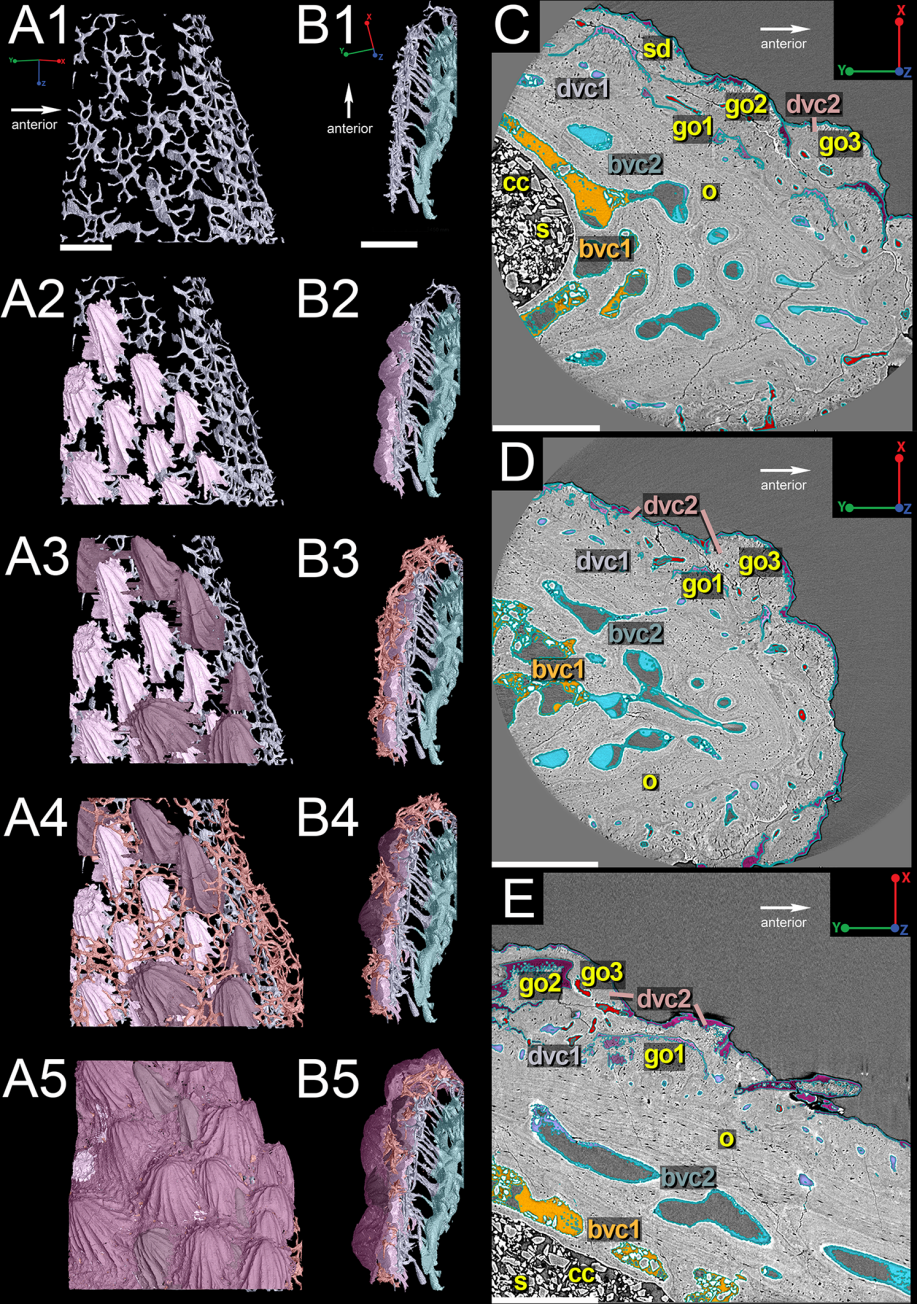
Organization of the *Romundina* dorsal ridge spine vascularization and odontodes. (A) Lateral and (B) cross-sectional views, along with virtual thin sections from high-resolution data (0.678 μm), illustrating how all parts appear in the slices taken in (C) transverse and (D) sagittal sections through the spine. Scale bars are 350 μm. Abbreviations are: bvc, bone vascular canals; cc, central canal; dvc, dentine vascular canals; go1, 2, 3, first, second, third generation odontodes; o, osteocyte spaces; s, sediment; sd, semidentine.

The two layers of dentine vascular canals (dvc1 and dvc2) are separated from the vascularized bone by long, narrow canals (Fig 4B), and are positioned where the ascending canals in the fin spine of *Lophosteus* were identified [24]. These two layers are closely situated together and are characterized by clusters of homogeneously sized and shaped (rounded) narrow canals (Fig 4A; purple & red). The clusters of dentine vascular canals correspond to the three generations of odontodes (Fig 5)–dvc1 relates to the first and second generations of odontodes (Fig 5A1 & 5B1) while dvc2 correlates to the third generation of odontodes (Fig 5 A2 and 5B2).

## Discussion

Here, for the first time we are presenting the analysis of the three-dimensional architecture and growth mode of a dorsal ridge spine of *Romundina stellina*, produced by propagation phase-contrast synchrotron X-ray microtomography. This three-dimensional histological study provides a basis for comparison with dermal skeletal structures of both fossil and living vertebrates.

The *Romundina* spine described here is most similar to the median dorsal spine described by Goujet and Young [19]. It is symmetrical and spinous with a flared base with odontodes that are elongated distally and rounder proximally (Fig 1B and 1C). The most striking difference between this *Romundina* spine and the one described by Goujet and Young [19] is its much smaller size, in addition to the posterior side remaining "open" and the distal tip not coming to a point (Fig 1B4). The ornament pattern strongly supports that this spine is a juvenile stage of the fin spine, although we still need to investigate the histology of the adult spine using the same approach.

Large vascular pore openings on the posterior surface of a fin spine from the putative osteichthyan *Lophosteus* are present in the area where the fin attached and is confirmed by the presence of Sharpey's fibers along that same area [24]. Unfortunately, we cannot confirm this in *Romundina*, as we do not have high-resolution scan data for the parts of the spine that would most likely contain Sharpey's fibers (discussed in more detail in the next section). However, the spine was associated with soft tissue because of the high concentration of vascular canals opening on the ventral and posterior surfaces and the presence of the perichondral bone and its associated cartilage insertion.

### Histology of the spine compared to other dermal structures of *Romundina*

The paleohistology of parts of the dermal skeleton of *Romundina* have been described from thin sections [37] and synchrotron data [26,28,30], but there are none relating to the dorsal spine. Giles et al. [30] reported that the scale of *R*. *stellina* is composed of three layers, summarized here as: (1) a basal layer of lamellar bone that is infiltrated with Sharpey's fibers and very few cell lacunae [also, 26], (2), a median layer of spongy osteonal bone with many osteocyte spaces and layers of polarized fibers dispersed throughout the bone matrix and sometimes exhibiting spheritic mineralization, and (3), a superficial layer of semidentine odontodes capped by a single crystalline enameloid monolayer (as defined by Gillis and Donoghue [38]). A similar outer layer of enameloid has been identified in other synchrotron datasets of *Romundina* material by Rücklin & Donoghue [29]. However, Burrow et al. [39] demonstrate that the outer layer in thin sections is not birefringent under polarized light and the dentine tubules penetrate through the entire layer, calling into doubt this enameloid identification.

Overall, our synchrotron data for the spine differs slightly from the scale histology described by Giles et al. [30]. We identify three layers, including 1) a thinner basal bone layer with no osteons and few cell lacunae, 2) a middle bone layer composed only of osteonal bone and many cell lacunae, and 3) an ornamented outer layer composed of multi-generations of semidentine odontodes capped with a possible hypermineralized tissue of unknown nature (Fig 2C). The middle layer of tissue in the spine shows no evidence for spheritic mineralization or multiple layers of fibers, but instead has zones of poorly mineralized bone tissue (Fig 2B; yellow arrowheads). Zones similar to these are observed in the tip of the spine of *Lophosteus* [24], but are proportionately less frequent in *Romundina* based on the available data.

The thin concave portion connecting the lateral faces of the posterior side of the spine appears to be composed of perichondral bone. Sharpey's fibers have not been observed in this dataset but have been observed from synchrotron data of the dermal bones and scales of placoderm taxa [30]. The basal layer is also vascularized but no osteons are present around the canals and there are very few cell lacunae and no Sharpey's fibers (Fig 2B). If Sharpey's fibers are present in the spine, they would be, 1), present in the perichondral bone around the ovoid opening on the proximal surface of the spine (Fig 1B4 and 1D), and, 2) within the perichondral bone along the length of the posterior surface and possibly the inside surface of the lateral flanks that face it (Fig 1). Unfortunately, there is no high-resolution synchrotron data of this region to use as confirmation.

The *Romundina* spine data described here is also different with regards to the outer layer of enameloid described by Giles et al. [30] and identified by Rücklin & Donoghue [29,40] (Fig 2B). While we observe a change in tissue density distally on each semidentine odontode (Fig 2C), we cannot confirm the presence of enameloid on the spine. Although Rücklin & Donoghue [29,40] compare their enameloid layer with the single crystalline enameloid in primitive chondrichthyan teeth, the single occurrence of this tissue in *Romundina* does not support such comparison since there is no phylogenetic continuity of the two tissues. A closer examination of thin sections of a dermal bone from *Romundina* suggests that this distinct outer layer in scan data corresponds to a darker and yellowish layer in real thin sections (S1 Fig). The dentine tubules almost go through this layer to the surface (S1 Fig, arrowheads) and there are embedded cell spaces (S1 Fig, arrows), which are not observed in the outer hypermineralized tissue in chondrichthyans (enameloid) or actinopterygians (acrodin). With embedded cell spaces, thin tubules and a higher density compared to the dentine below, this tissue remains enigmatic and cannot be compared with known mineralized tissues; thus it is not considered as enameloid following Burrow et al. [39].

### Growth of the dermal spine of *Romundina*

Publications detailing the ontogenetic data for the dorsal fin spines of the extant chondrichthyans *Callorhinchus* [16] and *Squalus* [15,16] create a valuable comparative context for the dorsal spine of *Romundina*. Even though the tomographic data presented here provide only a static image of an adult spine, the three-dimensional organization of the hard tissues and in particular the vasculature and odontode arrangement allow us to draw robust and detailed conclusions about its mode of growth, which can be compared with the data from *Callorhinchus* and *Squalus*.

**Fig 6:**
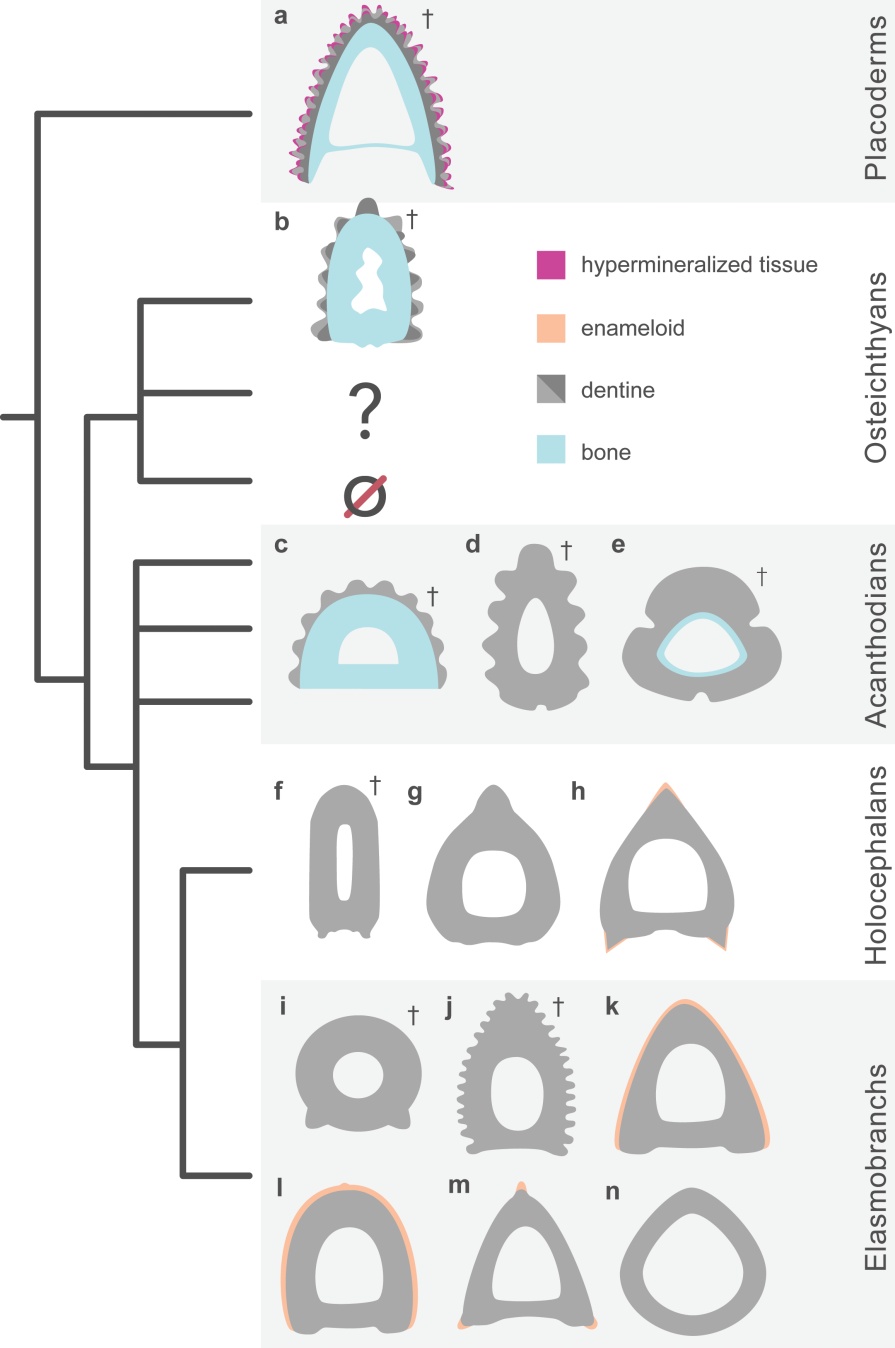
A generalized distribution of bone, dentine, and enameloid in the dorsal ridge spine of (a) *Romundina* and median dorsal fin spines from other fossil and non-fossil material. Several examples are given to show the morphological differences in cross-section. Examples are: (b), *Lophosteus* [24]; (c), *Nostolepis* [23,32], (d), *Diplacanthus* [10]; (e) ischnacanthid ([23,32]; (f), *Ischyodus* [41]; (g) *Chimaera* [41]; (h), *Callorhinchus* [16]; (i), *Orthacanthus* [42,43]; (j), *Ctenacanthus* [16,22]; (k), *Squalus* [15,16]; (l), *Heterodontus* [15]; (m), *Etmopterus* [15]; and, (n), *Oxynotus* [15]. The question mark indicates extinct taxa that bear fin spines but currently have no histological data and the null sign indicates the loss of dermal fin spines in crown osteichthyans. Daggers indicate extinct taxa.

All three spines have one feature in common, namely the presence of a central cartilage (observed in *Callorhinchus* and *Squalus*, inferred from the presence of perichondral bone in *Romundina*). Furthermore, in all three taxa the dermal biomineralized component has its greatest extent along the anterior margin of this cartilage. In all three, the proximal part of the spine is open posteriorly; however, in the two chondrichthyans the distal part of the spine is closed posteriorly and surrounds the cartilage, whereas in *Romundina* the spine is open posteriorly all the way to the tip. In other respects, the spine of *Romundina* is very different from the other two, as regards composition (Fig 6), three-dimensional organization and growth mode.

The chondrichthyan spines are composed entirely of dentine, which can be subdivided into three components [15,16]. The outermost of these is mantle dentine, which forms the apex of the spine and all or part of its exposed surface (it is more extensive in *Squalus* than in *Callorhinchus*). This is a conventional dentine that begins to be deposited in the contact surface between an epithelial fold and the underlying mesenchyme, and then grows inwards; in effect, the entire mantle dentine can be regarded as a very large odontode with its apex at the spine tip. The innermost component is trunk dentine, a laminated tissue that begins to form inside a mesenchymal condensation of odontoblasts and then grows both outwards and inwards. The trunk dentine is deeply inserted into the mantle dentine cone in *Squalus*, but joins the mantle dentine almost edge-to-edge in *Callorhinchus*. External to the trunk dentine, and in the distal part of the spine enclosed between the mantle and trunk dentines, *Callorhinchus* has an extensive development of trabecular dentine, a tissue consisting of dentine deposited in the form of osteon-like "denteons" around the blood vessels of a vascular plexus. This tissue is common and extensively developed in Palaeozoic chondrichthyans [22,41,44] but is absent in *Squalus* and other modern spine-bearing sharks [15,16].

In *Romundina*, the bulk of the spine consists of dermal bone, covered on the outside with three generations of small odontodes plus associated bone of attachment, which merges with the semidentine layer. There is no equivalent of the single large distal odontode represented by the mantle dentine of chondrichthyans. The growth center of the spine is a splint-like region that occupies the distal 40% or so of the leading edge (Fig 3). New material has subsequently been added posteriorly (i.e., towards the trailing edge, making the spine wider) and proximally. This contrasts with the chondrichthyans, where the early establishment of the spine tip (defined by the epithelial fold of the bud that generates the mantle dentine) prevents any further widening of the distal part and means that all extension growth occurs at the proximal end of the spine. The multiple generations of odontodes on the spine of *Romundina* contrasts with the single generation of accessory odontodes added along the posterolateral margins of the spine in *Callorhinchus* [16]. In summary, the growth modes of the *Romundina* spine on the one hand, and the chondrichthyan spines on the other, are quite different. The spine of *Romundina* appears, in essence, to be a conventional dermal bone, whereas the spines of *Squalus* and *Callorhinchus* are complex dentine structures dominated by single, extremely large, apical odontode (the mantle dentine).

The histological and microanatomical data from a median dorsal fin spine of the probable stem-osteichthyan *Lophosteus* that was recently presented by Jerve et al. [24] covers only the tip of the spine and thus cannot be fully compared with the spine data from *Romundina*, despite being produced by the same technique. *Lophosteus* is known only from isolated remains [45,46] and several different types of spine have been associated with the genus. Some of the *Lophosteus* fin spines are broadly similar in appearance to that of *Romundina*. The scan data revealed that the spine of *Lophosteus* also consisted of dermal bone carrying several generations of odontodes that have been added in a manner very similar to *Romundina* [24], suggesting that these spines were covered with epithelium that allowed repeated initiation of odontode formation. Differences are also apparent, most importantly the fact that the *Lophosteus* spine is closed posteriorly and carries a distinct posterior surface containing a mesh of Sharpey's fibers, probably indicating the attachment of the associated fin. Nevertheless, it is clear from these data that the spines of *Romundina* and *Lophosteus* are more similar to each other than to the extant chondrichthyans, *Squalus* and *Callorhinchus*, in addition to many fossil total group chondrichthyans in terms of morphology, histological composition, and growth. Such similarity further highlights the homology of dermal skeletal system between placoderms and osteichthyans [2,3]. However, more 3D data are needed from other parts of the median spine of *Lophosteus* for better comparisons between them and *Romundina*.

## Conclusions

We have shown that studying the 3D histology of fossils can provide new and important information about the development in extinct animals. Our data from this dorsal ridge spine of *Romundina* illustrates, for the first time, the complexity of its microstructure, which is broken down into two discrete layers of bone and multiple generation of dentine odontodes. Additionally, we have identified four layers of vascularization, two of which are responsible for the deposition of bone and two responsible for the deposition of dentine. Lastly, our data show that the dermal spine grew quite differently from the chondrichthyans *Squalus* and *Callorhinchus*. The compositional and growth differences between these fin spines demonstrates the need for studying those from other jawed vertebrates using 3D data, in particular, the acanthodians, whose spines can be composed of bone and/or dentine and other early osteichthyans that bear spines, such as *Psarolepis*. Fin spine composition from the handful of published histological studies shows that some acanthodian spines can be composed of bone and dentine, similar to both *Romundina* and *Lophosteus*, but that the proportions of these tissues are reversed, with dentine comprising the main part of the spine. Since acanthodians have been placed as stem chondrichthyans in recent phylogenetic analysis [2,3,12, 31], they may thus help to bridge the gap between the chondrichthyan and *Romundina*-*Lophosteus* spine types, and clarify the homology relationships between them (refer to [32]).

## Supporting information

S1 Fig Thin section (S2368) of a dermal plate of *Romundina*, deposited at the Natural History Museum of Stockholm. Arrows mark the cell lacunae and arrowheads mark the dentine tubules. Note the different colors of the outer layer and the inner layer, both of which have dentine tubules and cell lacunae.
(TIF)
