# Supplementary figures and images for: Vascularization and odontode structure of a dorsal ridge spine of Romundina stellina Ørvig 1975
Source: PLoS One. 2017 Dec 27;12(12):e0189833. doi: 10.1371/journal.pone.0189833 (PMC5744956; doi:10.1371/journal.pone.0189833)

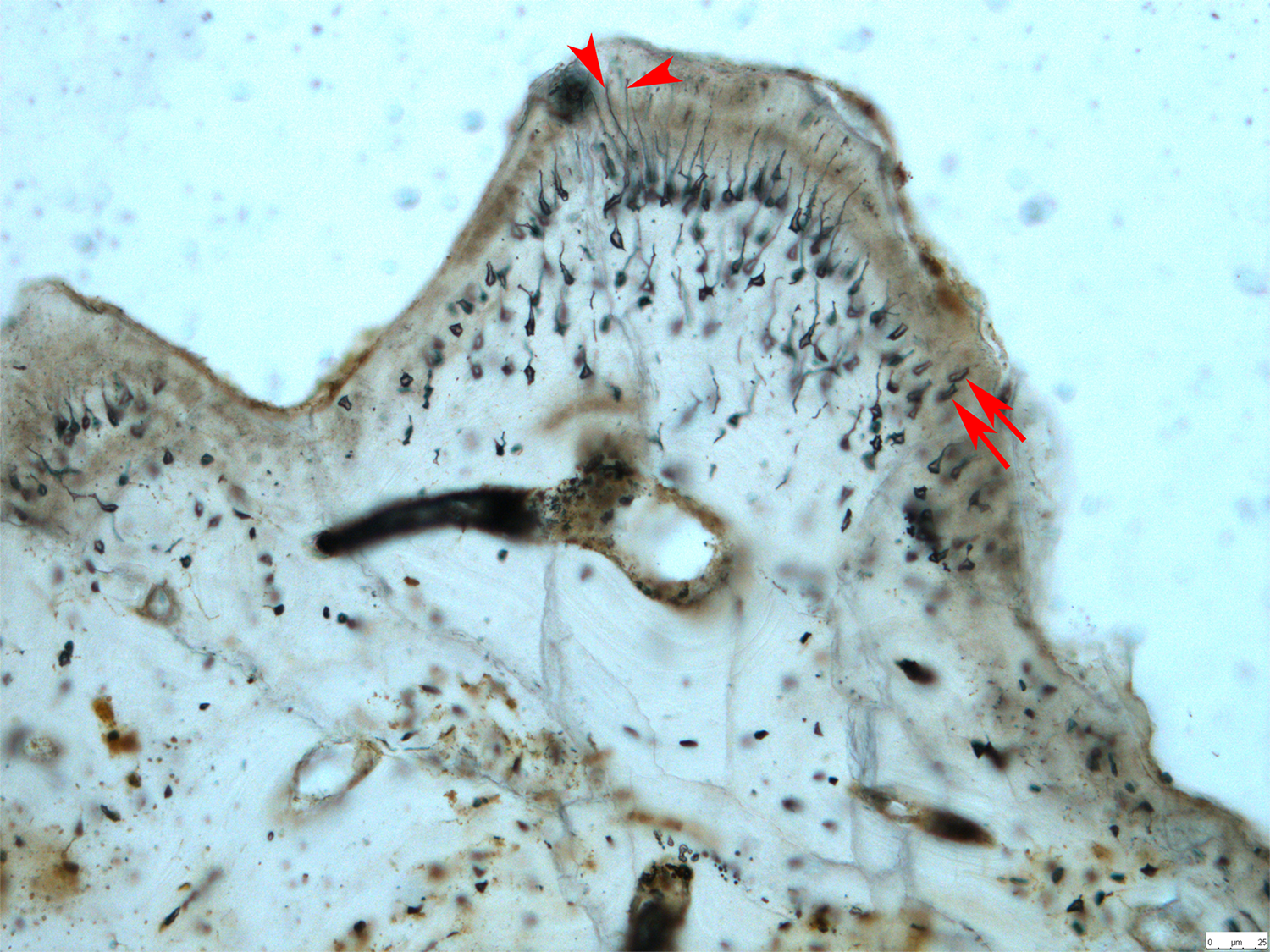

Supplement: S1 Fig — Arrows mark the cell lacunae and arrowheads mark the dentine tubules. Note the different colors of the outer layer and the inner layer, both of which have dentine tubules and cell lacunae. (TIF) [file pone.0189833.s001.tif]
